# Filling Teeth for Children

**Published:** 1848-07

**Authors:** 


### FILLING TEETH FOR CHILDREN.

For a child ten years of age, take one ounce best Spanish glue; one ounce best loaf sugar. Make them into "jujube paste." When soft, fill the open covities in the teeth. Keep the fillings dry until hard when they will be "fit for use." The great benefit in this mode of filling, is, that it answers all the purposes of candy; costs much less; will last much longer, and will be likely to give a distaste for sweets, as it will in most cases, make the teeth ache.

